# Editorial: Novel technologies targeting the rehabilitation of neurological disorders

**DOI:** 10.3389/fnins.2024.1367286

**Published:** 2024-03-26

**Authors:** Jie Jia, Jingchun Guo, Lin Yao, Dingguo Zhang

**Affiliations:** ^1^Department of Rehabilitation Medicine, Huashan Hospital, Fudan University, Shanghai, China; ^2^National Clinical Research Center for Aging and Medicine, Huashan Hospital, Fudan University, Shanghai, China; ^3^National Center for Neurological Disorders, Shanghai, China; ^4^State Key Laboratory of Medical Neurobiology, MOE Frontier Center for Brain Science, Department of Translational Neuroscience of Shanghai Jing'an District Centre Hospital, Institutes of Brain Science, Fudan University, Shanghai, China; ^5^College of Computer Science, College of Biomedical Engineering and Instrument Science, Zhejiang University, Hangzhou, China; ^6^Department of Electronic and Electrical Engineering, University of Bath, Bath, United Kingdom

**Keywords:** neurological disorders, stroke, TMS, close-loop, novel technologies

## 1 Background

The Research Topic—Novel technologies targeting the rehabilitation of neurological disorders was launched to collect the latest research and progress of new rehabilitation technologies for neurological diseases. Finally, 20 papers were included, comprising 14 original articles, four reviews, and two research protocols. They cover many new technologies for the assessment and treatment of neurological diseases. The evaluation techniques include motor-evoked potential (MEP), magnetic resonance imaging (MRI), electroencephalogram (EEG), sensory-evoked potential (SEP), and functional near-infrared spectroscopy (fNIRS). Regarding treatment techniques, they are mainly divided into non-invasive brain stimulation techniques, such as transcranial magnetic stimulation (TMS), transcranial direct current/alternating current technology (tDCS/tACS), ultrasound technology, and mirror therapy (MT). In contrast, invasive brain stimulation techniques include the brain-computer interface (BCI), vagus nerve stimulation (VNS), deep brain stimulation (DBS), and Contralateral Seventh Cervical Nerve Transfer (CC7). However, the papers included in this Research Topic did not involve new techniques of invasive brain stimulation. In this editorial, we will provide a summary of the papers included in our Research Topic from three perspectives. We will also expand on some of the content that our Research Topic lacks.

## 2 Novel assessment methods

With the progress of brain science and technology, innovative techniques have been promoted and applied based on new detection principles, and more accurate assessment methods have promoted the rehabilitation of functional disorders caused by neurological diseases. This part covers the exploration of a variety of new assessment techniques in neurological rehabilitation.

### 2.1 fNIRS

As a relatively new imaging method, fNIRS is a non-invasive optical imaging technology based on the principle of neurovascular coupling. Wang et al. used fNIRS as a monitoring and evaluation basis to monitor the degree of increased metabolic activity in the prefrontal cortex of stroke patients, and meta-analysis showed the value of PFC research in exploring the double-task effect of stroke. Chen H. et al. collected the topological structure of the frontal functional network during resting state for analysis using fNIRS. The study aimed to explore changes present in the frontal functional network region of patients in the lowest conscious state. In addition, Lacerenza et al. used fNIRS as a detection method to measure the functional activation of the motor cortex during arm lifting.

### 2.2 TMS-EEG

The physiological mechanism of TMS is to generate a brief magnetic field by placing an electromagnetic coil on the scalp, which stimulates the activity of neurons in the cerebral cortex. EEG provides a non-invasive way of recording electrical activity in the brain. It measures the electrical activity of neurons in the cerebral cortex by placing multiple electrodes on the scalp. The technique of TMS with EEG can observe the cortical reactivity and connectivity, and record the changes of cortical excitability and connectivity evoked by TMS (Hernandez-Pavon et al., 2023).

### 2.3 fMRI

fMRI is one of the most widely used non-invasive functional imaging techniques in observing the efficacy of new technologies in the rehabilitation of neurological diseases (Feng et al., 2021). It can indirectly characterize the function of brain neurons by capturing changes in blood oxygen levels. Its greatest advantage is its high spatial resolution, which allows us to observe deep brain structures, which fNIRS and EEG do not possess (Varley et al., 2020). It can help us analyze and understand the way the human brain works in basic cognitive processes (such as attention, memory, sensation, and perception) and higher cognitive processes (such as language, problem-solving, reasoning, etc.), and explore the psychophysiological mechanisms of various brain diseases (Kim et al., 2017).

### 2.4 Respiratory ultrasound

Respiratory ultrasound is widely used, feasible, non-invasive, and convenient for clinical application. In terms of rehabilitation treatment, in addition to the observation of the lungs by ultrasound, it can also evaluate the activity and morphological measurement of the respiratory muscle of patients through ultrasound visualization, improve the information about the structure and function of the respiratory muscle, and have good reproducibility. Sufficient sensitivity to detect clinically important changes. Under our Research Topic, Liu, Yang et al. summarized and described the application of ultrasonic measurement of respiratory muscle, and summarizes the function of respiratory muscle in stroke patients.

### 2.5 SEP

SEP is a commonly used neurophysiological test in clinical practice, and it is the most objective method to evaluate sensory pathways. The latent waveform of somatosensory evoked potentials may indicate the source of neurogenesis and is a highly sensitive neurophysiological indicator. Liu Y. et al. is based on somatosensory evoked potentials to explore the correlation between light touch and two-point discrimination measurements in the assessment of upper limb function.

## 3 Non-invasive brain stimulation techniques

### 3.1 TMS

Transcranial magnetic stimulation (TMS) is an adjunctive treatment for neurological recovery in disorders like stroke. It aids post-stroke recovery through neurogenesis, vascular regeneration, and anti-inflammatory actions (Zhou et al.). Clinically, TMS is used for improving motor functions, speech, swallowing, cognition, mood, spasticity, and pain in stroke patients (Zhou et al.). For post-stroke cognitive impairment (PSCI), excitatory TMS on the left hemisphere's DLPFC improves cognition, attention, and memory (Han K. et al.). Intermittent theta burst stimulation (iTBS), a novel mode of TMS, might be a more advantageous and convenient treatment than the classic rTMS for patients with PSCI (Han M. et al.). Recent research focuses on optimizing TMS efficacy. iTBS increases motor cortical excitability and enhances performance in task-specific activities (Goldenkoff et al.). A study on rTMS intensity revealed its impact on treatment efficacy, with high-frequency (25 Hz) rTMS demonstrating bidirectional effects on hippocampal plasticity, offering potential benefits for memory loss (Chen S. et al.).

### 3.2 tDCS/tACS

Transcranial direct current stimulation (tDCS) is a convenient and low-cost option for treatment, which modulates the neuronal activity in the cerebral cortex using constant, low-intensity direct current. For the first time, a recent study has confirmed that bilateral tDCS was a boost for the recovery of those suffering from Parkinsonian tremor (Zhang et al.). A network meta-analysis study found that cathodal tDCS was the most promising treatment option to improve ADL capacity in people with stroke (Elsner et al., 2017). Iodice et al. (2017) reviewed that motor (i.e., hand dexterity) and cognitive performances (i.e., attention and working memory) can be improved by applying rTMS or tDCS alone or in association with motor/cognitive training, for pain's treatment by using tDCS. However, it's also necessary to study the effects of tDCS in patients with disorders of consciousness (Estraneo et al., 2017). Compared with the direct current stimulation of tDCS, transcranial alternating current stimulation (tACS) synchronizes and modulates brainwave oscillations via the bidirectional current. It has been observed that patients with cerebellar ataxia (CA) benefited from this alternative treatment, suggesting that tACS is a promising NIBS technique for CA (Liu, Lin et al.).

### 3.3 TUS

Focused transcranial ultrasound stimulation (TUS) is a highly accurate NIBS modality, which can straightly focus low-intensity sound waves to deeper cerebral areas (Folloni, 2022). With the potential of a short and offline therapeutic program, TUS based on its advantages can be a prospective treatment for neurological diseases, like stroke and Parkinson's (Wang et al., 2020; Folloni, 2022; Wang Y. et al., 2022). Presently, it's still in the initial exploratory phase, whose mechanisms are yet to be explored for extensible application.

### 3.4 MT

Mirror visual feedback (MVF), also known as mirror therapy (MT), has been widely applied in clinical neurorehabilitation. However, the underlying neuro-mechanisms of MVF are still unclear. It was believed to encourage cortical reorganization in the brain, which plays a crucial role in functional recovery after stroke. Recently, a study has verified that embodiment via MVF could bring denser functional brain connectivity to healthy people, digging deeper into these unknown mechanisms. In addition, an intervention combining mirror visual with vibrotactile stimulus is developed as a prospective rehabilitation strategy, which can enhance the embodiment perception (Ding et al.).

### 3.5 BCI

Brain-computer interface (BCI) is a communication channel by delivers the signals from the central nervous system (CNS) to external computers or machines. At present, this advanced technique has been used in various fields, including clinical rehabilitation training programs, typing communication systems, robotics, etc (Wang et al., 2023). For meeting the users with different training requirements and functional states, a personalized BCI paradigm has been established and optimized in terms of design, development, evaluation, and application (Ma et al., 2023).

### 3.6 Closed-loop

**Figure 1:**
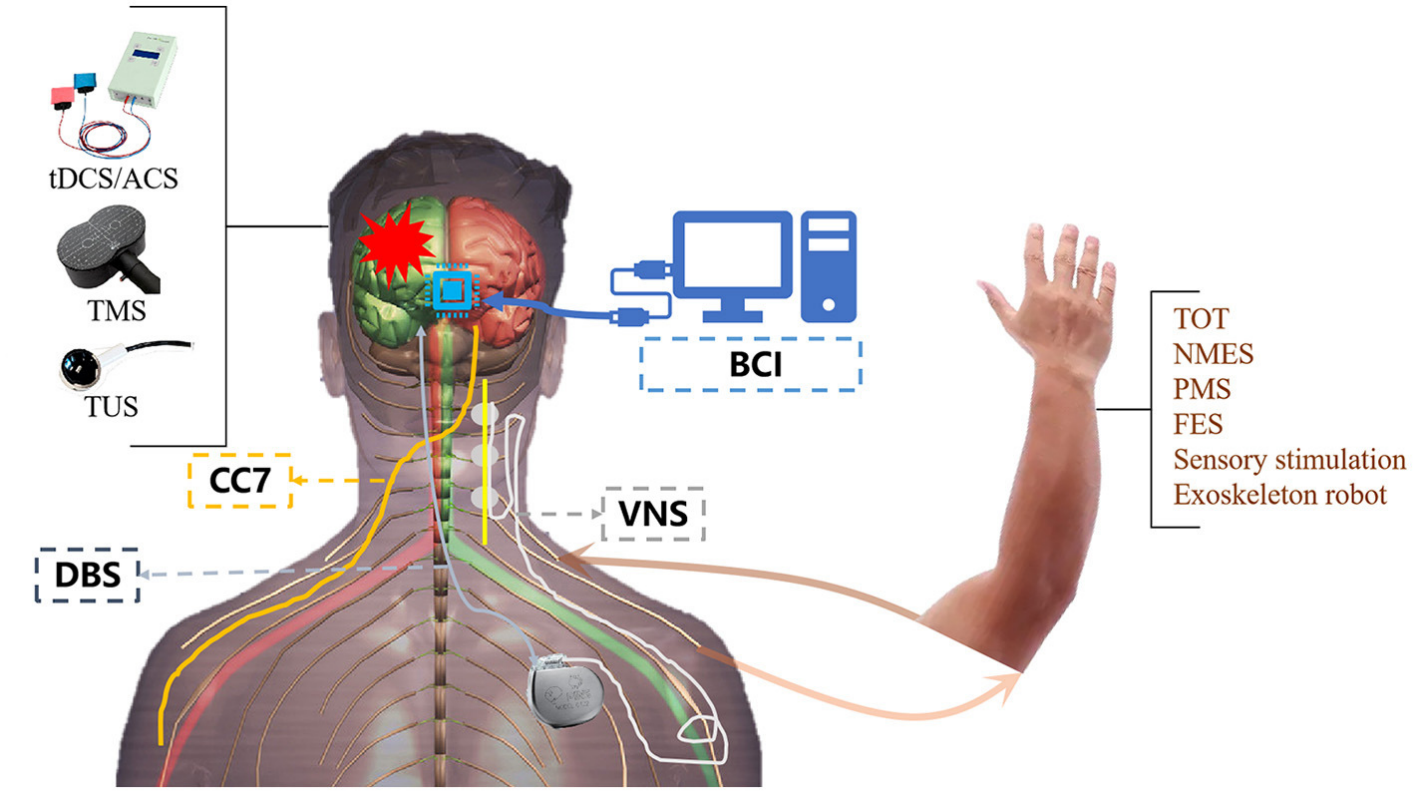
Central combined with peripheral stimulation techniques. TOT, task-oriented therapy; NMES, neuromuscular electrical stimulation; PMS, peripheral magnetic stimulation; FES, functional electrical stimulation.

Developed based on the central-peripheral-central concept, closed-loop rehabilitation is a therapeutic strategy that integrates the application of central and peripheral interventions (see Figure 1) (Jia, 2022). On behalf of the central intervention, non-invasive brain stimulation plays an important role in enhancing the links of corresponding brain networks and neuroplasticity (Yamamura et al., 2018). However, peripheral interventions like neurophysiological therapy reinforce the correct movement patterns through continuous sensory feedback. A novel closed-loop rehabilitation program, combining melodic intonation therapy (MIT) with tDCS verified its positive effects for post-stroke aphasia (Yan et al.). An Individualized closed-loop TMS system coordinated with an exoskeleton was developed to recover the patients with different spasticity states (Singh et al.).

## 4 Invasive techniques

Invasive brain stimulation has shown promising prospects in improving the dysfunction caused by organic or non-organic neurological diseases such as stroke, Parkinson's disease, and Alzheimer's disease. Invasive brain stimulation technology can have various options according to the stimulation depth, stimulation location, stimulation tools, and so on. At present, in the rehabilitation of neurological diseases, invasive brain stimulation technology has been widely concerned with invasive brain-computer interface technology (invasive BCI), deep brain stimulation technology (DBS), vagus nerve electrical stimulation technology (VNS), as well as the contralateral seventh cervical nerve transfer (CC7) surgery.

### 4.1 Invasive brain-computer interface (invasive BCI)

Invasive BCI records information about brain activity by surgically implanting electrodes in the brain that are placed very close to or directly on target neurons in targeted cortical regions or subcortical structures, allowing for neural signals with higher spatial resolution (Zhao et al., 2023). The effectiveness of invasive BCI has been extensively tested in animals like macaques, and in 2007, invasive BCI was found to help restore motor function in a paralyzed patient (Donoghue et al., 2007). Since then, human research on invasive BCI has been gradually carried out, replacing impaired motor functions with robotic arms (Hochberg et al., 2012). In addition, with the help of invasive BCI, it can also compensate for functional deficiencies such as walking and speech (Dawson et al., 2016; Willett et al., 2021). The safety of invasive BCI under minimally invasive surgical operations has also been verified, providing stronger evidence for further promoting the application of invasive BCI (Mitchell et al., 2023).

### 4.2 Deep brain stimulation

Deep brain stimulation, which generally covers a large number of neurons, currently plays an important role in the treatment of neurological diseases and neuropsychiatric disorders. Boraud found that high-frequency DBS can alleviate hand and foot convulsions and other motor symptoms of Parkinson's patients, and there are also studies showing that DBS can improve the sleeping quality of Parkinson's patients (Boraud et al., 1996; Castillo et al., 2020). The effectiveness of DBS for Parkinson's patients has not only been demonstrated in patients with different disease courses but it has also been shown not to fade over time (Malek, 2019).

### 4.3 Vagus nerve stimulation

The first human installation of an electrical vagus nerve stimulator was in 1988, and since then several studies have been conducted to observe the effectiveness, safety, and tolerability of this technology as a treatment, as well as the selection of stimulation frequency. Dawson et al. (2016) used VNS to treat upper limb function in patients with chronic stroke and found that after 6 weeks of intervention, patients in the experimental group showed significant improvement in FMA-UE score compared with the control group, proving that VNS is a feasible and effective stroke rehabilitation method. Five years later, Dawson et al. (2021) continued to supplement the research findings and conducted more rigorous studies with larger sample sizes to further verify it. VNS technology has also been shown to be an effective means to treat cognitive disorders, epilepsy, depression, and so on (Fisher et al., 2015; Reif-Leonhard et al., 2022; Wang L. et al., 2022).

### 4.4 Contralateral seventh cervical nerve transfer (CC7)

In 1986, Gu et al. (2002) pioneered the contralateral seventh cervical nerve transfer operation, which connects the cervical seventh nerve root in the healthy brachial plexus nerve with the nerve controlling the paralytic hand, and has been used to treat the patients with total brachial plexus nerve injury and has achieved remarkable results. So far, this technology has been applied for nearly 40 years, and has played an important role in promoting the recovery of upper limb hand function in stroke patients (Hong et al., 2019). In 2018, Zheng et al. used CC7 surgery to treat patients with chronic stroke and found that it can improve patients' upper limb motor function and relieve spasms (Zheng et al., 2018). The efficacy has been further verified through a multi-center, large-scale research in 2022, and a standardized guidance reference will be provided for the promotion and application of this technology (Feng et al., 2022).

## 5 Limitations

For research on innovation assessment methods, most sample sizes are small due to constraints on assessment sites and equipment. Small sample sizes and the limited information available made it difficult to analyze other confounding factors, such as injury types, injury regions, and different age groups. In particular, the clinical prediction Models require more sample sizes and multiple medical institutions for better clinical generalizing (Yu et al.). Some brain imaging assessment methods (e.g., EEG, fNIRS) use specific-channel caps, that might limit further exploration of sub-network alterations (Ding et al.). For clinical research of non-invasive brain stimulation techniques and invasive techniques, considering patients' limited hospital days and the difficulty of post-discharge follow-up, some researchers just focus on the short-term effect and pay less attention to the long-term effect (Zhang et al.; Liu, Lin et al.) (Dawson et al., 2021). Most clinical studies are performed in a single center, and they may not represent the results from other regions (Liu, Lin et al.), also cannot generalize the findings to people who do not meet trial eligibility criteria or to people with other types of stroke or other neurological disorders (Dawson et al., 2021). There are different conditions of one neurological disorder (e.g., different types, disease urgency, duration), so stimulation parameters used for the same neurological disorder may also vary from different studies, which makes the optimal treatment protocol difficult to find.

## 6 Conclusion

Nowadays, increasing research focuses on the use of new technologies in the rehabilitation of neurological disorders. The innovative assessment methods can complement existing assessment methods and provide in-depth functional assessments from neurophysiological or brain imaging perspectives, which may aid in better understanding the mechanism of neurological disorders. The innovative treatment methods show significant treatment effects in many studies. In the future, researchers involved in novel technologies targeting the rehabilitation of neurological disorders should recruit more samples from different institutions and regions to verify the stability and repeatability of the data. Additionally, more randomized controlled trials with large samples, high quality, and follow-up are needed to explore a usable protocol.

## Author contributions

JJ: Writing – original draft, Writing – review & editing, Supervision. JG: Supervision, Writing – review & editing. LY: Supervision, Writing – review & editing. DZ: Writing – review & editing.

## References

[B1] Boraud T. Bezard E. Bioulac B. Gross C. (1996). High frequency stimulation of the internal globus pallidus (GPi) simultaneously improves parkinsonian symptoms and reduces the firing frequency of GPi neurons in the MPTP-treated monkey. Neurosci. Lett. 215, 17–20. 10.1016/S0304-3940(96)12943-8 8880743

[B2] Castillo P. R. Middlebrooks E. H. Grewal S. S. Okromelidze L. Meschia J. F. Quinones-Hinojosa A.. (2020). Globus pallidus externus deep brain stimulation treats insomnia in a patient with parkinson disease. Mayo Clin. Proc. 95, 419–422. 10.1016/j.mayocp.2019.11.020 32029093

[B3] Dawson J. Liu C. Y. Francisco G. E. Cramer S. C. Wolf S. L. Dixit A.. (2021). Vagus nerve stimulation paired with rehabilitation for upper limb motor function after ischaemic stroke (VNS-rehab): a randomised, blinded, pivotal, device trial. Lancet 397, 1545–1553. 10.1016/S0140-6736(21)00475-X 33894832 PMC8862193

[B4] Dawson J. Pierce D. Dixit A. Kimberley T. J. Robertson M. Tarver B.. (2016). Safety, feasibility, and efficacy of vagus nerve stimulation paired with upper-limb rehabilitation after ischemic stroke. Stroke 47, 143–150. 10.1161/STROKEAHA.115.010477 26645257 PMC4689175

[B5] Donoghue J. P. Nurmikko A. Black M. Hochberg L. R. (2007). Assistive technology and robotic control using motor cortex ensemble-based neural interface systems in humans with tetraplegia. J. Physiol. 579, 603–611. 10.1113/jphysiol.2006.127209 17272345 PMC2151381

[B6] Elsner B. Kwakkel G. Kugler J. Mehrholz J. (2017). Transcranial direct current stimulation (TDCS) for improving capacity in activities and arm function after stroke: a network meta-analysis of randomised controlled trials. J. Neuroeng. Rehabil. 14:95. 10.1186/s12984-017-0301-7 28903772 PMC5598049

[B7] Estraneo A. Pascarella A. Moretta P. Masotta O. Fiorenza S. Chirico G.. (2017). Repeated transcranial direct current stimulation in prolonged disorders of consciousness: a double-blind cross-over study. J. Neurol. Sci. 375, 464–470. 10.1016/j.jns.2017.02.036 28320187

[B8] Feng J. Li T. Lv M. Kim S. Shin J. H. Zhao N.. (2022). Reconstruction of paralyzed arm function in patients with hemiplegia through contralateral seventh cervical nerve cross transfer: a multicenter study and real-world practice guidance. EClinicalMedicine 43:101258. 10.1016/j.eclinm.2021.101258 35028546 PMC8741478

[B9] Feng M. Wen H. Xin H. Zhang N. Liang C. Guo L.. (2021). Altered spontaneous brain activity related to neurologic dysfunction in patients with cerebral small vessel disease. Front. Aging Neurosci. 13:731585. 10.3389/fnagi.2021.731585 34975450 PMC8718906

[B10] Fisher R. S. Eggleston K. S. Wright C. W. (2015). Vagus nerve stimulation magnet activation for seizures: a critical review. Acta Neurol. Scand. 131, 1–8. 10.1111/ane.12288 25145652

[B11] Folloni D. (2022). Ultrasound neuromodulation of the deep brain. Science 377:589. 10.1126/science.add4836 35926020

[B12] Gu Y. Xu J. Chen L. Wang H. Hu S. (2002). Long term outcome of contralateral C7 transfer: a report of 32 cases. Chin. Med. J. 115, 866–868. 12123554

[B13] Hernandez-Pavon J. C. Veniero D. Bergmann T. O. Belardinelli P. Bortoletto M. Casarotto S.. (2023). TMS combined with EEG: recommendations and open issues for data collection and analysis. Brain Stimul. 16, 567–593. 10.1016/j.brs.2023.02.009 36828303

[B14] Hochberg L. R. Bacher D. Jarosiewicz B. Masse N. Y. Simeral J. D. Vogel J.. (2012). Reach and grasp by people with tetraplegia using a neurally controlled robotic arm. Nature 485, 372–375. 10.1038/nature11076 22596161 PMC3640850

[B15] Hong G. Liu J. Liu Y. Gao K. Zhao X. Lao J.. (2019). Modified contralateral C7 nerve transfer: the possibility of permitting ulnar nerve recovery is confirmed by 10 cases of autopsy. Neural Regen. Res. 14, 1449–1454. 10.4103/1673-5374.253530 30964072 PMC6524498

[B16] Iodice R. Manganelli F. Dubbioso R. (2017). The therapeutic use of non-invasive brain stimulation in multiple sclerosis – a review. Restor. Neurol. Neurosci. 35, 497–509. 10.3233/RNN-170735 28984619

[B17] Jia J. (2022). Exploration on neurobiological mechanisms of the central-peripheral-central closed-loop rehabilitation. Front. Cell. Neurosci. 16:982881. 10.3389/fncel.2022.982881 36119128 PMC9479450

[B18] Kim J. H. Yu D. Huh Y. H. Lee E. H. Kim H. Kim H. R.. (2017). Long-term exposure to 835 MHZ RF-EMF induces hyperactivity, autophagy and demyelination in the cortical neurons of mice. Sci. Rep. 7:41129. 10.1038/srep41129 28106136 PMC5247706

[B19] Ma Y. Gong A. Nan W. Ding P. Wang F. Fu Y.. (2023). Personalized brain–computer interface and its applications. J. Pers. Med. 13:46. 10.3390/jpm13010046 PMC9861730 36675707

[B20] Malek N. (2019). Deep brain stimulation in parkinson's disease. Neurol. India 67, 968–978. 10.4103/0028-3886.266268 31512617

[B21] Mitchell P. Lee S. C. M. Yoo P. E. Morokoff A. Sharma R. P. Williams D. L.. (2023). Assessment of safety of a fully implanted endovascular brain-computer interface for severe paralysis in 4 patients: the stentrode with thought-controlled digital switch (switch) study. JAMA Neurol. 80, 270–278. 10.1001/jamaneurol.2022.4847 36622685 PMC9857731

[B22] Reif-Leonhard C. Reif A. Baune B. T. Kavakbasi E. (2022). [Vagus nerve stimulation for difficult to treat depression]. Nervenarzt 93, 921–930. 10.1007/s00115-022-01282-6 35380222 PMC9452433

[B23] Varley T. F. Luppi A. I. Pappas I. Naci L. Adapa R. Owen A. M.. (2020). Consciousness and brain functional complexity in propofol anaesthesia. Sci. Rep. 10:1018. 10.1038/s41598-020-57695-3 31974390 PMC6978464

[B24] Wang L. Zhang J. Guo C. He J. Zhang S. Wang Y.. (2022). The efficacy and safety of transcutaneous auricular vagus nerve stimulation in patients with mild cognitive impairment: a double blinded randomized clinical trial. Brain Stimul. 15, 1405–1414. 10.1016/j.brs.2022.09.003 36150665

[B25] Wang P. Cao X. Zhou Y. Gong P. Yousefnezhad M. Shao W.. (2023). A comprehensive review on motion trajectory reconstruction for EEG-based brain-computer interface. Front Neurosci. 17:1138406. 10.3389/fnins.2023.1138406 37332859 PMC10272365

[B26] Wang Y. Li F. He M. Chen S. (2022). The effects and mechanisms of transcranial ultrasound stimulation combined with cognitive rehabilitation on post-stroke cognitive impairment. Neurol. Sci. 43, 4315–4321. 10.1007/s10072-022-05906-2 35141805

[B27] Wang Z. Yan J. Wang X. Yuan Y. Li X. (2020). Transcranial ultrasound stimulation directly influences the cortical excitability of the motor cortex in parkinsonian mice. Mov. Disord. 35, 693–698. 10.1002/mds.27952 31829467

[B28] Willett F. R. Avansino D. T. Hochberg L. R. Henderson J. M. Shenoy K. V. (2021). High-performance brain-to-text communication via handwriting. Nature 593, 249–254. 10.1038/s41586-021-03506-2 33981047 PMC8163299

[B29] Yamamura K. Kurose M. Okamoto K. (2018). Guide to enhancing swallowing initiation: insights from findings in healthy subjects and dysphagic patients. Curr. Phys. Med. Rehabil. Rep. 6, 178–185. 10.1007/s40141-018-0192-y 30147997 PMC6096907

[B30] Zhao Z. P. Nie C. Jiang C. T. Cao S. H. Tian K. X. Yu S.. (2023). Modulating brain activity with invasive brain-computer interface: a narrative review. Brain Sci. 13:134. 10.3390/brainsci13010134 36672115 PMC9856340

[B31] Zheng M. X. Hua X. Y. Feng J. T. Li T. Lu Y. C. Shen Y. D.. (2018). Trial of contralateral seventh cervical nerve transfer for spastic arm paralysis. N. Engl. J. Med. 378, 22–34. 10.1056/NEJMoa1615208 29262271

